# Burden of Serious Fungal Infections in Argentina

**DOI:** 10.3390/jof4020051

**Published:** 2018-04-24

**Authors:** Fernando O. Riera, Juan P. Caeiro, David W. Denning

**Affiliations:** 1 Infectology Department, Sanatorio Allende Córdoba, Av Hipólito Yirgoyen 384, Córdoba, Argentina; 2 Infectious Diseases Section, Hospital Privado Universitario de Córdoba, Av. Naciones Unidas 346, Córdoba, Argentina; 3 The Natonal Aspergillosis Centre, Wythenshawe Hospital, Manchester Academic Health Science Centre, and The University of Manchester, Manchester M23 9LT, UK

**Keywords:** mycoses, epidemiology, aspergillosis, candidemia, fungal infections

## Abstract

The number of fungal infections at any given time in Argentina is not known. Here we estimate the burden of serious fungal infections in Argentina for the first time. Specific population statistics were searched from multiple sources, local literature was identified, and estimates made. Some additional data were sourced from the Ministry of Health, the Global Initiative for Asthma (GINA) program, and national haematology and transplant societies. Argentina has a population of 43.8 million, with 25% of this total being children under 15 years. The predicted candidemia annual incidence is 2193 cases, with 50% occurring in the ICU. At a 6% prevalence rate, an estimated 593,695 women suffer from recurrent vulvovaginal candidiasis. Invasive aspergillosis is relatively common because of high smoking and chronic obstructive pulmonary disease (COPD) rates, with 268 cases in immunocompromised patients and another 1938 in the 168,000 COPD patients admitted to hospital. Asthma is also common, affecting 14% of adults, and so allergic bronchopulmonary aspergillosis (ABPA) and severe asthma with fungal sensitization (SAFS) are major problems. An estimated 432 cases of cryptococcal meningitis (CM)—90% of them in AIDS patients—and 1177 cases of *Pneumocystis* pneumonia (PCP) occur each year. The estimated annual case number of disseminated histoplasmosis is 404 in AIDS patients, almost as frequent as CM. Paracoccidioidomycosis annual incidence is estimated at 219, and coccidioidomycosis at 16 cases. At least 881,023 people (>2.01%) in Argentina are affected by a serious fungal disease annually, with considerable morbidity and mortality.

## 1. Introduction

Argentina is the eighth-largest country in the world and the second-largest country in Latin America. It has a wide range of climates and several endemic fungal diseases are present. The Gross Domestic Product per capita is $12,000, and the average life expectancy is 77.1 years [1].

Fungal disease in humans ranges from trivial (tinea pedís between the toes) to usually fatal (cerebral aspergillosis and mucormycosis). In this paper, we have adopted the term “serious fungal disease” to encompass those entities that are often fatal (especially if the diagnosis is missed and treatment not given) as well as those with substantial morbidity such as fungal keratitis, tinea capitis, recurrent vulvovaginal candidiasis (rVVC), allergic fungal sinusitis, and fungal asthma (allergic bronchopulmonary aspergillosis (ABPA) and severe asthma with fungal sensitization (SAFS)).

As fungal diseases are not reportable, exact data are not available and the current number of fungal infections occurring each year in Argentina is not known. In addition, in Argentina there are few published papers on the incidence and prevalence of some life-threatening fungal disease entities. However, it is desirable to integrate new data to provide an overall estimate on the total population affected by fungal diseases in healthcare for the purposes of future research and healthcare planning. Diagnostic treatment gaps are also highlighted by comparing local data with those of other neighboring countries where estimates have been published, such as Brazil, Chile, Peru, and Ecuador [2,3,4,5,6].

The aim of this study was to estimate the total population in Argentina affected by serious fungal disease. By using the available epidemiological data, we have calculated the incidence and prevalence; for those diseases with no existing local data, we have used published data from neighboring countries, or international sources. We have applied calculations based on national, regional, and international cohort studies; clinical trials; and rates of infections already documented in similar groups of patients, as others have reported [2,3,4,5,6].

## 2. Materials and Methods

In order to elucidate the serious fungal burden in Argentina, we performed an exhaustive search of epidemiological reports. To estimate the fungal burden, data were retrieved from the National Institute of Statistics and Census (INDEC), the Annual Report of HIV and AIDS, National Administration of Laboratory and Health Institutes (ANLIS), and from the Ministry of Health publications. We also searched the bibliography for Argentinian data on mycotic diseases, asthma, Chronic Obstructive Pulmonary Diseases (COPD), cancer, and transplant patients. The incidence and prevalence for each fungal disease were estimated in specific populations at risk. When no data existed, risk populations were used to estimate frequencies of fungal infections, using the previously described methodology by Leading International Fungal Education (LIFE) [7].

The number of patients with human immunodeficiency virus (HIV)/acquired immune deficiency syndrome (AIDS) was obtained from epidemiological surveillance of AIDS in Argentina published in the Joint United Nations Programme on HIV/AIDS (UNAIDS) [8] and from the Argentina Annual Report of HIV and AIDS [9,10]. Similarly, the proportions of AIDS patients presenting with *Pneumocystis* pneumonia (PCP), *Candida* esophagitis, or cryptococcal meningitis in 2017 were calculated using estimates based on a literature review of manuscripts addressing the incidence rates of infections for a specific set of patients. PCP is most commonly associated with the initial presentation of AIDS in 25% of cases [11].

In a recent study, the prevalence of cryptococcal antigenemia in Argentina was 8.1% in those with CD4 counts of <100 × 10^6^/mL, and cryptococcal meningitis affected 4.87% of patients with AIDS in this series [12]. Furthermore, of 2041 patients with cryptococcosis, 98% of the cases were associated with AIDS and meningitis was seen in 90% of the patients [13].

In most populations, it is assumed that 90% of patients with late-stage HIV who are not being treated with highly active antiretroviral therapy (HAART) will develop oral candidiasis [14,15]. Earlier data from a US cohort study showed that oral candidiasis may occur in 16% of cases with HIV/AIDS treated with HAART [16]. Esophageal candidiasis was based on the assumption that 20% of HIV patients with CD4 counts under 200 × 10^6^/mL who are not on antiretroviral therapy are affected by this condition, as well as 5% of those on therapy [17,18].

Cases of candidemia in hospitalized patients were based on the prevalence rates documented between November 2008 and October 2010 in three tertiary care hospitals in Argentina as part of a multicenter study in Latin America [19]. The incidence of candidemia per 1000 admissions was 1.95 (range 1.26–2.98) and the incidence per 1000 patient-days was 0.24 (range 0.13–0.19). We assumed that 50% of candidemia episodes occur in the intensive care unit (ICU) and that the rate of *Candida* peritonitis is ~50% of the ICU candidemia rate [20,21,22,23].

The number of tuberculosis (TB) cases was taken from the epidemiological surveillance registry in the Ministry of Health of Argentina [24]. It is assumed that 22% of patients with lung cavities and 2% of those without cavities following pulmonary tuberculosis (PTB) will develop chronic pulmonary aspergillosis (CPA) [25]. Patients with PTB are expected to represent ~20% of the total number of CPA cases annually, so the total prevalence of CPA from any cause is estimated using the national PTB figures [26].

Asthma rates in Argentina were obtained from the first prevalence study by the Ministry of Health of Argentina. In 2015, 5.98% of the adult population were estimated to have asthma [27]. The risk of allergic bronchopulmonary aspergillosis (ABPA) was estimated at 2.5% based on previous studies from other countries [28]. The rate of severe asthma with fungal sensitization (SAFS) was estimated for the most severe 10% of cases of the total asthma population; out of that, 33% were assumed to have fungal sensitization [29,30,31].

An estimated 2.3 million people in Argentina are living with a diagnosis of COPD, a prevalence of 14.5% in those >40 years old. We assumed that 7% of these patients are admitted to hospital each year [32].

Argentina’s cancer data were reported by the International Agency for Research on Cancer through the Globocan Project in 2012, showing an estimated incidence of crude rates for leukemia at 5.1/100,000; acute myeloid leukemia (AML) is the most common type of leukemia in adults [33]. Overall, there were 2372 cases of leukemia reported, and 11,244 people were diagnosed with lung cancer in 2012.

It is assumed that non-acute myeloid leukemia hematological conditions in total represent the same population incidence of invasive aspergillosis (IA) as do AML patients—an incidence of approximately 10% [34]. Furthermore, it is assumed that the incidence of IA in allogeneic hematopoietic stem cell transplants is 8% [35].

IA is also associated with solid organ transplantation, although the reported incidence varies by both dataset and anatomical site. The Transplant-Associated Infection Surveillance Network data found the 1 year cumulative incidence of documented IA to be 0.5% of renal, 2.0% of heart, 0.9% of liver, and 9.1% of lung transplants [36]. Given the size of this dataset, it provides the most precise disease rates and we have adopted these values. Argentinian transplant data were obtained from the 2016 Instituto Nacional Central Único Coordinador de Ablación e Implante (INCUCAI) report on organ transplant and organ donation registry. In Argentina in 2016, 1687 solid organ transplants were performed [34]. Of the total 1687 transplants, 1121 were renal (740 with deceased donor and 381 with living donor), 349 were livers (312 with deceased donors and 37 with living donors), 109 were hearts, and 33 were lungs. In addition, 937 corneal transplants were performed [37]. We have assumed that the rate of IA in lung cancer patients was 2.6%, as described by Yan et al (2009) [38].

Recurrent *Candida* vaginitis (more than 4 episodes per year) was included in the context of serious infections due to its impact on quality of life as well [39]. For recurrent *Candida* vaginitis, the number of cases was based on 6% expected prevalence among women of ages 15 to 50 years old [7,40]. According to the INDEC, the population of this age group of women in Argentina is ~9,894,900.

Mucormycosis was estimated using data forma population studies in which incidence was 1.7 million inhabitants and data from Transplant-Associated Infection Surveillance Network (TRANSNET) [41,42].

In 2004, the National Network of Mycology Laboratories of Argentina (ANLIS) reported the results of a retrospective nationwide survey on mycoses diagnosed between January and December, 2004. The study included data provided by 72 laboratories located in 19 provinces of Argentina. A total of 23,600 mycoses cases were diagnosed, of which 1663 patients (7%) presented with deep mycoses. Although the survey is based on laboratory data only, we used these data to estimate the incidence per 100,000 patients according to the population of that year (38,730,000) and we apply that indicator to the current population [43].

## 3. Results

Argentina has a population of approximately 43.8 million, with 25% of these being children under 15 years old and 16% being women >60 years old. The average life expectancy is 77.1 years [1]. Demographic data of the Argentinian population are presented in Table 1, including the main at-risk categories for fungal diseases. The gross domestic product was USD $12,450 per capita in 2016.

### 3.1. Infections in HIV-Infected Patients

In Argentina there are 120,000 people living with HIV/AIDS and 6500 new cases are notified annually; 27.5% of these people are diagnosed late, so they often have or are at immediate risk of acquiring an opportunistic infection [10].

The annual incidence of cryptococcosis is difficult to establish because cryptococcosis is not a reportable disease in Argentina. In a study of HIV-positive patients ≥18 years of age with advanced immunosuppression, CrAg-positivity was 8.1% [10,11]. The estimated incidence is 0.85/100,000 cases or a total of 372 cases in AIDS, with another 60 cases in non-HIV patients. Microbiologic confirmation of PCP diagnosis is rarely accomplished in most developing countries. PCP prevalence among adult HIV-infected patients ranges widely, from 5% to 60%, due to use of different diagnostic techniques. The estimated incidence is 2.68/100,000, a total of 877 in AIDS, with another 300 cases in non-HIV patients. The estimated number of oral and oesophageal candidiasis cases in patients with HIV is 7200 cases (Table 2).

### 3.2. Histoplasmosis

In 1945, the Argentinian mycologist Dr. Pablo Negroni published the first case of histoplasmosis in South America [44]. Histoplasmosis is the most frequent endemic mycosis in Argentina—30–40% of apparently healthy adult inhabitants of endemic areas have been infected by *H. capsulatum* [45]. Most of the reported cases correspond to the region known as Pampa Húmeda that includes the provinces of Buenos Aires, Entre Ríos, Santa Fe, Córdoba and La Pampa. A large part of the country’s population is concentrated in this area. Several outbreaks have also been described in the country [45,46]. AIDS patients account for about 90% of documented cases. Disseminated histoplasmosis has been reported in 5.3–6% of advanced HIV infection patients in Argentina with frequent skin involvement, followed by pulmonary disease [47]. Radiological findings include miliary patterns, interstitial infiltrates, and focal infiltrates [48]. The estimated incidence based in laboratory data is 0.45/100,000 assuming that this represents only 50% of the diagnoses [43,49]. We estimated 404 cases annually. No estimate is provided for subacute or chronic forms of histoplasmosis.

### 3.3. Endemic Fungal Infections Other Than Histoplasmosis

Paracoccidioidomycosis (PCM) has a restricted geographic distribution. It is endemic in humid subtropical areas of Latin America, from Mexico to Argentina [45]. In Argentina, there are two endemic areas—one in the northeast and one in the northwest [50]. In endemic areas, a population prevalence of 7.16% has been reported [51]. In recent years, the frequency of cases of PCM has increased, but with different epidemiological characteristics to those historically reported, with more patients presenting with the juvenile clinical form [52]. The estimated incidence is 0.5/100,000—an estimated 219 cases annually.

Coccidioidomycosis was reported for the first time in Argentina in 1892 by Posadas and Wernicke [53]. Clinical cases of coccidioidomycosis are rare in Argentina and are generally found in the large arid precordilleran area of the country [54]. The incidence rate is estimated at only 2/100,000 inhabitants in the endemic area (Catamarca, La Rioja, and San Luis provinces), with an estimated 16 cases [51].

### 3.4. Candida Infections Other Than Those Seen in HIV-Infected Patients

The incidence of bloodstream infections per 100,000 in Latin America varies annually between 1.2 and 5.3. Data from hospitals in Argentina showed an incidence rate of 1.95/100,000 admissions and 0.24/1000 patients in hospital [19]. *Candida albicans* was the most common species (52.2%), followed by *C. parapsilosis* (30.4%) and *C. tropicalis* (10.9%) [23]. The estimated number of cases of candidemia was 2193 and 548 of *Candida* peritonitis.

In a review of oral fungal infections in patients receiving cancer therapy, for all cancer treatments, the weighted prevalences of clinical oral fungal infections were found to be 7.5% pretreatment, 39.1% during treatment, and 32.6% after the end of cancer therapy [33]. These rates may differ each year but may be used as an estimate for current calculations. Each year, 55,000 new cancer cases (including hematological cancers) are recorded. Assuming that the majority of cancer patients (>90%) receive anti-cancer treatment, oral candidiasis episodes were calculated, resulting in a total of 23,375 episodes and 632 oesophageal cases each year.

It was assumed that 6% of women have recurrent vaginal candidiasis (four or more episodes per year), which correlates with 593,695 Argentinian women with recurrent vaginal thrush in any one year.

### 3.5. Chronic, Allergic, and Invasive Aspergillosis

Of the 10,423 cases of TB in 2016, mostly in HIV-negative people, 8891 (85.3%) had pulmonary tuberculosis. There were 757 deaths and we have assumed that all of them were pulmonary TB cases, leaving 8134 survivors. Using the approach taken by Denning et al. which assumed a 22% cavitation rate following therapy, with a 22% rate of CPA in this group and a 2% in the remainder, we estimate that annually at least 469 patients develop CPA following pulmonary TB, which gives a 5 year period prevalence of 1938, assuming a 15% annual mortality and surgical resection rate. It was assumed that TB was the underlying diagnosis of CPA in 20% of cases and so the total CPA prevalence is 7750 patients [24,25,26].

Estimates of asthma prevalence in adults were around 6% [27]. Assuming that 2.5% of asthmatics have ABPA, there will be 114,709 Argentinian adults with ABPA (261/100,000) [28,29]. In fact, there are no reports of ABPA from Argentina or Latin America, so this estimate is uncertain. Severe asthma is also an increasing problem in Argentina, and fungal allergy has been documented in Brazil, although not in Argentina. Nonetheless, we have assumed that 10% of adults have severe asthma and 33% of these people have fungal sensitization, so we would expect about 151,416 (345/100,000) to have severe asthma with fungal sensitization (SAFS) [30]. There may be some duplication between these prevalence figures, as some patients with ABPA have severe asthma.

According to the Ministry of Public Health of Argentina, 2.3 million people have chronic obstructive pulmonary disease (COPD), and it is assumed that 7% of these patients are admitted to hospital [32]. Given that 1.3–3.9% of these patients develop invasive aspergillosis, we anticipate at least 1934 such cases annually, although many are not currently diagnosed. Recently, over 10% of patients with COPD were found to be sensitized to *A. fumigatus* and this was associated with worse pulmonary function; therefore, we expect 230,000 patients with COPD to have allergic or chronic aspergillosis, although the therapeutic implications of this are not clear [32].

The most important risk factor for IA has historically been neutropenia, and we estimate 480 cases of IA in leukemia patients. According to the INCUCAI data in Argentina, there were 1687 transplants. We estimate the incidences of IA in 22 cases after allogeneic hematopoietic stem cell transplant and in 13 cases after solid organ transplantation. In addition, IA causes complications in patients with lung cancer. A retrospective study reported a frequency of 2.6% [38]; we estimate 268 cases of IA complicating lung cancer. The total IA incidence is estimated at 2536.

We estimated 75 cases of mucormycosis, most in immunocompromised patients.

Overall, it is estimated that ~881,023 people in Argentina (2.01% of the total population) are affected by a serious fungal infection. We were not able to estimate the burden of other serious fungal diseases such as, mycetoma, chromoblastomycosis, sporotrichosis, fungal keratitis, or tinea capitis.

## 4. Discussion

This is the first attempt to summarize epidemiological data on the prevalence of serious fungal diseases in Argentina and has used a basic deterministic model. There are few epidemiology papers that have directly reported incidence, prevalence, or fungal infection rates, so every estimate is based on previously published data. As George Box [55] wrote in a statistics workshop proceedings paper published in 1979, “All models are wrong but some are useful” and “Now it would be very remarkable if any system existing in the real world could be exactly represented by any simple model. However, cunningly chosen parsimonious models often do provide remarkably useful approximations.” This has been our aim.

Our overall estimate of 2.01% of the total population affected by a serious fungal infection is similar to others reported for American countries such as Brazil, Dominican Republic, Jamaica, Mexico, Trinidad, Tobago, Peru, and Ecuador ranging from 1.9% to 2.4% [35,56]. It is similar to the rates reported for some European developed countries, such as Denmark and Belgium (1.7% and 2.1%, respectively), and for African countries such as Senegal and Tanzania (1.6% and 2.3%) [56]. The estimated rate for Argentina is also lower than the ones reported from Spain and Germany (3 and 3.6%) [56].

Recurrent *Candida* vaginitis is the most frequent infection and usually affects women without known underlying diseases; it is the most frequent indication for using antifungals and is therefore, a potential source for colonization by azole-resistant *Candida* strains.

We believe that the 2.01% figure is an underestimation of the real burden of serious fungal diseases in Argentina, with no true explanation for cause, other than missing data. First, mycotic diseases are underdiagnosed due to inadequate access to health services, the limited sensitivity of available diagnostic tests, and an inadequate level of clinical suspicion of fungal diseases explained by inadequate training of health personnel. This is a common problem across the world. These reasons could explain the small number of endemic mycoses diagnosed in Argentina, even though one-third of the territory has conditions for acquisition of them. The perception of a small burden is one reason why flucytosine, a critically important and WHO Essential Medicine, has not been available for more than two decades in Argentina, as in most Latin American countries.

The second reason for believing that we have an underestimated burden is that reporting of fungal diseases is not mandatory and there is no surveillance system for any mycotic disease.

Third, the lack of data available to estimate the size of populations at risk drove us to use conservative figures.

This first estimation of the fungal burden in Argentina needs to be refined, improving measures of fungal diseases and of populations at risk. We acknowledge that using this data representing the country as a whole, and using data not locally produced, has the potential for introducing substantial levels of inaccuracy. The use of better data will produce better estimations and allow trend analyses. However, our results create awareness on fungal diseases that are a public health priority.

## Figures and Tables

**Table 1:** Demographic data for the calculation of fungal-related diseases in Argentina.

Population estimates in July 2016	43,886,748
**HIV and AIDS estimates**	
People living with HIV 2016	120,000
New HIV infections	6500
AIDS-related death	2400
HIV + on highly active antiretroviral therapy (HAART)	75,400
Patients at risk (CD4 count <200 and who developed AIDS)	1105
% Late diagnosis (AIDS symptoms and <200 CD4) at risk of opportunistic infection (OI)	2275 (35%)
**Leukemia, transplant, and other immunocompromised patients**	
Leukemia	1369
All cancers excl. non-melanoma skin cancer	54,953
Solid organ transplant 2016 year	1687
Renal	1121
Liver	349
Heart	109
Pancreas	46
Lung	33
Liver–renal	17
Intestinal	4
Heart–renal	4
Pancreatic–liver	2
Liver pulmonary	1
Heart–hepatic	1
**Pulmonary diseases**	
Tuberculosis total	10,525
Pulmonary tuberculosis (PTB)	9605
Annual incidence	26,3/100,000
HIV-positive TB patients	525
Lung cancer	7690
Chronic obstructive pulmonary disease	2,300,000
Prevalence (for patients over 40 years old)	14.5%
Chronic obstructive pulmonary disease admissions to hospital per year	168,000
**Asthma**	
In adults >40 years old	4,588,360
**Cystic fibrosis**	
Total registered	156
**Critical care and surgery cases (2013 year)**	
Critical care beds	9116

**Table 2:** Burden of serious fungal infection in Argentina.

	Rate/100,000	Burden						
Serious Fungal Infection			Totals	No Underlying Disease	HIV/AIDS	Respiratory Disease	Cancer + immuno-Compromised	Critical Care + Surgery
Cryptococcal meningitis	1.0	I	432		372		60	
Pneumocystis pneumonia	2.68	I	1177		877		300	
Invasive aspergillosis	5.8	I	2536			268	334	1934
Chronic pulmonary aspergillosis post TB	1.1	I	615			615		
Chronic pulmonary aspergillosis post TB	4.0	P	1938			1938		
Chronic pulmonary aspergillosis—all	19.9	P	7750			7750		
Allergic bronchopulmonary aspergillosis (ABPA)	261	P	114,709			114,709		
Severe asthma with fungal sensitisation (SAFS)	345	P	151,416			65,172		
Candidaemia	5.0	I	2193				1096	1097
*Candida* peritonitis	1.25	I	548					
Oesophageal candidiasis	13.1	I	5743		5111		632	
Recurrent *Candida* vaginitis (≥4×/year)	2706	P	593,695	593,695				
Mucormycosis	0.17	I	75	13			62	
Disseminated histoplasmosis	0.91	I	404	37	367		40	
Coccidioidomycosis	0.03	I	14	14				
Paracoccidioidomycosis	0.56	I	246	246				
Total serious fungal infection burden			881,023					
			2.01%					

I: incidence, P: prevalence.
